# Which patient benefit most from minimally invasive direct anterior approach total hip arthroplasty in terms of perioperative blood loss? A retrospective comparative study from a cohort of patients with primary degenerative hips

**DOI:** 10.1007/s12306-023-00792-z

**Published:** 2023-06-14

**Authors:** M. Brunello, A. Di Martino, F. Ruta, R. Ferri, V. Rossomando, C. D’Agostino, D. Pederiva, F. Schilardi, C. Faldini

**Affiliations:** 1 1St Orthopaedic and Traumatologic Clinic, IRCCS Istituto Ortopedico Rizzoli, Via G.B. Pupilli 1, 40136 Bologna, Italy; 2 Department of Biomedical and Neuromotor Science-DIBINEM, University of Bologna, Bologna, Italy

**Keywords:** Total hip arthroplasty, Direct anterior approach, Posterolateral approach, Blood loss, Anticoagulant, Antiaggregant, Outcomes

## Abstract

**Introduction:**

Total hip arthroplasty (THA) is a successful surgery, but despite the advancements in anesthesiology and orthopedics, sometimes blood transfusions are required to manage the anemia due to the blood loss, involving a substantial number of patients. The aim of this retrospective comparative study is to define how the choice of the surgical approach, either direct anterior (DA) or posterolateral (PL), may influence the postoperative blood loss and the need for transfusion in THA.

**Materials and methods:**

Data collection was carried out retrospectively of THAs performed between 2016 and 2021 on primary hip osteoarthritis treated by DA or with PL approach. Clinical and perioperative anesthetic data were collected. Preoperative hemoglobin levels were compared with the lowest detected level by calculating ΔHb (hemoglobin decrease). Then, data from the two groups were cross-checked: duration of surgery, whether premedication with tranexamic acid, duration of the hospitalization, rate of need for hemotransfusions, and amount of blood transfused. The two samples were subdivided into subgroups according to age, BMI, tranexamic acid prophylaxis, and chronic treatment with drugs that alter coagulative properties.

**Results:**

Time of surgery was longer for patients treated with DA access (mean DA: 78.8 min; mean PL: 74.8 min; *p*: 0.05; 95% CI), but the length of hospitalization was shorter for patients treated with DA group with a mean time of 6.23 days versus 7.12 days for the PL group (*p* < 0.01). DA THA resulted advantageous mainly in patients between 66 and 75 years, showing a reduced postoperative transfusion requirement in the postoperative period (DA: 13.43%—mean: 1.33 units; PL: 26.82%—mean: 1.18 units; *p*: 0.044, 95% CI). Patients that assume blood-altering drugs showed a higher transfusion rate (*p* < 0.01), but comparison of the two subgroups showed that the choice of the surgical approach did not significantly affect the transfusion rate in these patients (*p*: 0.512). Prophylaxis with tranexamic acid reduced the transfusion rate (*p* < 0.01).

**Conclusion:**

Patients treated by minimally invasive direct anterior approach undergo a significantly shorter hospitalization. From the analysis of patient’s subgroups those aged between 66- and 75-years benefit from the DA approach mainly for the minor blood loss with less frequent transfusion requirement.

## Introduction

Total hip arthroplasty (THA) is one of the most effective treatments in major orthopedic surgery to treat effectively end-stage degenerative arthritis of the hip with groin pain and joint loss function. This surgery can achieve optimal long-term outcomes and acceptable rates of complications: For these reasons THA has been defined as "the operation of the century" [1].

THA surgery gained notoriety for the long-term implant survival and for the elevated functional performances that patients can habitually obtain with this joint replacement. However, THA is associated with a low but non-negligible risk of perioperative complications, ranging from minor adverse such as postoperative anemia to major complication until risk of death [2].

Moreover, the physiological consequence of surgery can lead to blood loss that could determine an anemia condition if not adequately managed during the procedure.

Despite the progresses in anesthesiology and orthopedics, blood transfusions are often required to manage the anemia due to blood loss: With an estimated perioperative transfusion rate averaging 18% up to 26%, this could represent an additional cost to health cares [3–5].

Blood loss involves directly the patient’s outcome, and it is clinically proven that patients with perioperative blood loss show longer in-hospital stay and slow postoperative rehabilitation [6]. Bleeding is related to an increased risk to develop postoperative hematoma, that can raise the risk of postoperative pain and development of infection, and may require revision surgery [7].

The success of the surgery in terms of reduction of blood loss and complication rate depends on a skillful coordination by the surgeon and anesthetist: The implementation of minimally invasive surgical techniques and appropriate perioperative drug protocols is meant in this direction [8, 9].

Tranexamic acid, epsilon-amino-caproic acid and aprotinin are the most studied drug protocols used to support a facilitated recovery after THA. The use of tranexamic acid, an antifibrinolytic agent that inhibits the activation of plasminogen, demonstrated effects through intravenous, oral and topical administration [10, 11] and significantly supports the reduction in transfusion rate to as low as 10% [12].

The minimally invasive DA approach to the hip has recently gained popularity for its ability to speed up the rehabilitation process and to shorten the duration of hospitalization [13].

The purpose of this retrospective study is to analyze the impact of surgery and patient-related factors on blood loss and requirement of blood transfusion in the perioperative setting, analyzing two cohorts of patients operated on primary THA, respectively, through minimally invasive DA or PL approach at the Authors’ Institution.

## Materials and methods

Current study and all the cases collected have been approved by the Local Ethical Committee (CE-AVEC) with the code 021 ANT-HIP, 347/2021/Oss/IOR. Patients were retrospectively considered for inclusion if they were operated for primary THA from January 1, 2016, to December 31, 2021 at the Authors’ Institution. Inclusion criteria were: Patients affected by primary osteoarthritis and treated by THA through DA and PL approach, with a follow-up of at least 12 months. Exclusion criteria were: Patients treated with different approaches than DA and PL, undergoing bilateral hip arthroplasty, with secondary post-traumatic osteoarthritis, congenital hip dysplasia, femoral neck fracture, suffering from osteochondrosis or other diagnoses different from primary hip osteoarthritis. All the procedures were performed by 5 expert senior surgeons of the unit. The average follow-up was 24 months. At the acetabular level all the patients were implanted by VERSAFIT-Cup or MPACT-Cup (Medacta International, Swiss); at the femur site, implants were AMIStem (Medacta International, Swiss). Patients were subsequently divided into 2 groups according to their surgical approach: direct anterior (DA) and posterolateral (PL) approach.

### Demographic and clinical information

Demographic and clinical parameters were retrospectively collected from the medical records of the hospital: age at surgery, American Society of Anaesthesiologists (ASA) score, surgical time (from surgical incision to end of suture), in-hospital length of stay, chronic treatment with anticoagulants and antiplatelet agents, information regarding other associated internal pathologies, perioperative laboratory data included Hb (mg/dL) and Hct (%) collected preoperatively and periodically during the in-hospital stay, need for blood transfusions reported as total amount of transfused blood components. Any intra- or postoperative complications were recorded, while in-hospital and at outpatient evaluations performed at 1, 3 and 6 months postoperatively.

### Pharmacological therapy

Patients under treatment with oral anticoagulants (AC) and antiplatelet (AP) agents suspended their administration before surgery according to the institution corporate protocol for elective surgery (Tables 1 and 2):

**Table 1:** Anticoagulants institution corporate suspension protocol for elective surgery

	Hours before surgery (h)	INR
Dabigatran	24–48	1.0–1.5
Apixaban	24–48	1.0–1.5
Rivaroxaban	24–48	1.0–1.5
K vitamin antagonist	120 h–bridging with LMWH	1.5–2.0

When neuraxial anesthesia was required in procedures with a high risk of bleeding, an additional 24 h were added to the suspension times

**Table 2:** Antiaggregant institution corporate suspension protocol for elective surgery

	Days before surgery
Cardioaspirin	Continues (if in primary prevention)
Clopidogrel	5–7 days
Ticlopidine	10 days
Ticagrelor	3–5 days
Prasugrel	7–10 days
Indobufene	2 days
Dipyridamol	1–2 days

After surgery, all patients have performed prophylaxis of enoxaparin sodium at a prophylactic dose of 50 UI/Kg. For the statistical analysis, the decreasing of hemoglobin was considered from the difference between preoperative values and the postoperative minimum recorded value (ΔHb).

Target for blood transfusions usually was below 8 mg/dL, with each transfusion consisting of 300 mL of homologous concentrated red blood cells per unit. Using the same statistical tools, subgroups were identified according to age (< 55 years, > 55 years and < 65 years, > 65 years and < 75 years, > 75 years); other subgroups include patients on chronic anticoagulation or antiaggregation, patients who was administered tranexamic acid during perioperative period, and obese patients (BMI > 30).

Study variables were analyzed and compared among groups. Comparison was made using Student’s T tests and Chi-square tests (SPSS 14.0, version 14.0.1; SPSS Inc, Chicago, IL). Significance was set at *p* value < 0.05.

## Results

From a total of 931 primary THAs performed from January 1, 2016, to December 31, 2021, 647 patients (335 males and 312 females; 52% M and 48% F) matched the inclusion criteria and were considered for analysis.

The overall population was divided by approach with 500 patients that were included in DA group, 257 (51%) males and 243 (49%) females, while 147 were in PL group, including 78 (53%) males and 69 (47%) females. According to BMI, there was an overall prevalence of obesity in 25% (160/647) patients: In specific 125 were in DA (25%), and 35 (24%) in PL group.

Patients under treatment with anticoagulants or antiaggregants were 118 (24%) from DA group and 45 (31%) from PL group. In the DA group 87 (17%) patients were treated with antiaggregants and 31 (6%) patients with anticoagulant; they suspended their treatment following the Institution’s protocol. In the PL population 37 (25%) took antiaggregant drugs, while 8 (5%) anticoagulants.

Surgical time registered showed that PL group had lower duration compared to DA, with an average time of 74.8 (range: 21 min – 165 min) minutes compared to the 78.8 (range: 28 min – 142 min) minutes for DA group (p value: 0.05; 95% CI). The obese patients required a further longer operative time [obese: 80.70 min (range: 33 min – 165 min); non-obese: 76.86 min (range: 38 min – 123 min)] (p value: 0.048; 95% CI) (Table 3).

**Table 3:** Summary of demographic characteristics divide by approach

Population	Direct anterior approach group	Posterolateral approach group
Total	500	147
Male/female	257 (51%)/243 (49%)	78 (53%)/69 (47%)
Age	Average: 64 yo (range: 34–95)	Average: 66 yo (range: 35–88)
Anticoagulants & Antiplatelet ag	118 (24%)	45 (31%)
Anticoagulants	31 (6%)	8 (5%)
Antiplatelet agents	87 (17%)	37 (25%)
Obese patients	125 (25%)	35 (24%)

### Postoperative hemoglobin

The mean decrease in hemoglobin (ΔHb) values in the two groups was comparable without difference (mean: DA: 4.0 mg/dl; PL: 4.0 mg/dl) (*p* value: 0.732), as for the comparison between patients in treatment with anticoagulants and not, which had results comparable to the normal population (VM: 4.0 mg/dl).

The comparison of the two groups according to stratification by age group (< 55 years, 56–65, 66–75 years, > 76 years) did not give difference (DA < 55 aa: 4.14 mg/dl; PL < 55 aa: 3.75 mg/dl; *p* value: 0.247); (DA 56–65 aa: 3.87 mg/dl; PL 56–65 aa: 4.00 mg/dl; *p* value: 0.552); (DA 66–75 aa: 4.16 mg/dl; PL 66–75 aa: 7.13 mg/dl; *p* value: 0.074) (DA > 76 aa: 7.57 mg/dl; PL > 76 aa: 7.19 mg/dl; *p* value: 0.655) (Table 4).

**Table 4:** Summary of hemoglobin decrease divided by approach

ΔHb	Direct anterior approach group (mg/dl)	Posterolateral approach group (mg/dl)	*p* value
Whole sample	4.0	4.0	0.732
< 55 yo	4.14	3.75	0.247
56–65 yo	3.87	4.00	0.552
66–75 yo	4.16	7.13	0.074
> 76 yo	7.57	6.19	0.655

The evaluation of patients treated with tranexamic acid showed a less blood loss compared to those who not received the treatment, regardless of the approach (ΔHb with Tranex: 3.61 mg/dl; ΔHb without Tranex: 4, 13) (*p* value: 0.00001).

Obese patients performed by DA showed a less blood loss in Hb in the postoperative period compared to patients operated through the PL approach, but without difference for statistical significance (obese DA: 3.95 mg/dl; obese PL: 4.35 mg/dl; *p* value: 0.074).

### Frequency rate of allogeneic blood transfusions in the postoperative days

Considering two groups, there was no difference in the number of transfusions (DA: 17.40%; PL: 20.41%) (*p* value: 0.404), and also the number of transfusion in both groups did not show any difference in blood unit (DA: 1.41 units, range DA: 1–5 units; PL: 1.47 units, range PL: 1–3 units).

The population was stratified by age group, and we identified that the 14.88% of patients under 55 years, treated by DA, were transfused, while in Pl group the 13.04% (DA: 1.50; PL: 1.33) (*p* value: 0.819). Patients between 56 and 65 years in 12.50% of cases, treated by DA approach, were transfused, compared to 8.70% of patients treated by the PL approach (AD: 1.42; PL: 1.75) (*p* value: 0.480). In the 13.43% of patients aged 66 to 75 years, treated by DA, were transfused, compared to 26.82% of patients treated with PL (AD: 1.33; PL: 1.18) (*p* value: 0.044; 95% CI).

Patients over 76 years in 34.40% of cases, in DA group, were transfused, while in PL group the 32.43% (AD: 1.41; PL: 1.67) (*p* value: 0.830; 95% CI) (Table 5).

**Table 5:** Summary of transfusion rate divided by approach

Transfusion rate	Direct anterior approach group	Posterolateral approach group	*p* value
Whole sample	17.40%—Average 1.41 units	20.41%—Average1.47 units	0.404
< 55 yo	14.88%—Average 1.50 units	13.04%—Average 1.33 units	0.819
56–65 yo	12.50%—Average 1.42 units	8.70%—Average 1.75 units	0.480
66–75 yo	13.43%—Average 1.33 units	26.82%—Average 1.18 units	0.044
> 76 yo	34.40%—Average 1.41 units	32.43%—Average 1.67 units	0.830

### Analysis of subgroups

From the analysis of two groups, a sub-analysis was carried out by evaluating parameters like anticoagulant or antiplatelet therapy, obesity and peri-operative pharmacological premedication (Table 6).

**Table 6:** Summary of transfusion rate divided by approach and independent factors

Transfusion rate	Direct anterior approach group	Posterolateral approach group	*p* value
With tranex	17.40%—Average 1.41 units	20.41%—Average 1.47 units	0.404
Anticoagulants/antiplatelet ag	34.18%—Average 1.55 units	36.36%—Average 1.50 units	0.512
Anticoagulants	14.88%—Average 1.50 units	13.04%—Average 1.33 units	0.819
Antiplatelet agents	12.50%—Average 1.42 units	8.70%—Average 1.75 units	0.480
Obese	16.00%—Average 1.33 units	11.43%—Average 1.18 units	0.521

#### Tranexanemic acid administration

Patients who received premedication with tranexamic acid were 144 (22%), of these 126 were treated with DA approach while 18 with PL approach. The comparison of all patients undergoing tranexamic acid prophylaxis versus those who did not receive this treatment showed that this treatment is effective in reducing rate of postoperative blood transfusions (Tranex: 7.75%; No Tranex: 20.99%) (*p* value: 0.0003) and it is advisable in all patients, especially patients with high risk of bleeding, while no difference was shown in the use of tranexamic acid between DA and PL group.

#### Treatment with blood drugs

Patients on chronic treatment with antiplatelet (AP) drugs or anticoagulants (AC), reported more frequently blood transfusions compared normal population (AC or AP: 31.01%; No AC and AP: 13.91%) (*p* value: 0.00001), transfusing on average 1.53 units and 1.35 units of blood, respectively. Patients on chronic oral antiplatelet treatment required transfusions more frequently (32.26%) than patients who do not (14.72%) (*p* value: 0.00001), while patients on chronic treatment with anticoagulants required more frequently blood transfusions (28.21%) compared to patients not subjected to this treatment (17.43%), but without a statistical difference (*p* value: 0.0902).

Analyzing subgroups of patients in AC and AP treatment, the comparison between DA and PL did not show difference about transfusion rate in either of the two subgroups (AP and AD vs. AP and PL: *p* value 0.711) (AP and AD vs. AP and PL: *p* value 0.486).

#### Obese patients

Obese patients required a longer operative time compared to a not-obese patients, independently by the surgical approach (obese: 80.70 min; not-obese: 76.86 min) (*p* value: 0.048). The bleeding parameters did not show difference in blood loss in the comparison between obese and not-obese patients (ΔHb obese: 4.04 mg/dl; ΔHb not-obese: 4.00 mg/dl) (*p* value: 0.521); the same results came out from the comparison of obese patients treated by DA and PL approach (ΔHb AD: 3.95 mg/dl; ΔHb PL: 4.35 mg/dl) (*p* value: 0.227).

## Discussion

The study emerges that in a population between 65 and 75 years old who performed THA by DA approach the transfusion rate is lower compared to PL group, but other interesting results show that the anterior approach may be beneficial in some patients, such as the obese patient.

The results show that the application of the minimally invasive anterior approach to the hip guarantees, compared to the posterolateral, a short-term hospitalization (DA: 6 days, PL: 7 days) and this result is common by other authors like Petis et al. [14] in his review, he showed that the mean length of stay in hospital was 2.28 days for the direct anterior group and 3.02 days for the PL group. In a comparative study by Nakata et al. [15] recorded a mean length of stay of 30.4 days for posterior approach and 22.2 days for direct anterior approach (*p* = 0.003). There are similar results that we found in the study by Poehling-Monaghan et al. [16], where the author reported the effectiveness of DA approach in early functional benefits compared to a mini- posterior approach. The aforementioned results with different length of stay in order of days have to be related with postoperative rehabilitation protocol, where hospitalization included also a partial or a total rehabilitation.

Another parameter that has to be indagated is the surgical time, the minimally invasive anterior approach to the hip is discussed by authors, our results show a longer surgical time for DA compared to PL approach. In accordance, we found the meta-analysis by Cha et al. [17] showed a significantly longer operation time for DA compared to PL and also Parvizi et al. found significantly longer operating time compared to posterolateral approach associating the prolonged operative time with increased blood loss [18].

Our manuscript showed that there are no significant differences in the postoperative decrease in hemoglobin (ΔHb) in primary total hip replacement performed by DA compared to the posterolateral approach. The literature is varied in this topic, in the meta-analysis by Yang et al. [19] that has been evaluated 467 cases in DA group and 465 cases in the PA group: with a minimal difference in terms of mean blood loss with a mean value of 5.63 ml (*p* value: 0.88). Miller et al. [20] in a meta-analysis included 609 patients from RCTs documented no significant mean blood loss when comparing the two approaches, anterior and posterolateral (mean difference = 19 mL, *p* = 0.71). In contrast prospective randomized study by Cheng et al. [21] that recruited 75 matched patients undergone to DA or PLA (38 vs. 37) by expert surgeons, reports a greater blood loss with the use of the anterior approach compared to the posterolateral one (*p* value: 0.04), recognizing an independent risk factor for bleeding in the longer surgical time.

The frequency for use of transfusions has been shown to be lower in patients aged 66 to 75 years. These data show how adult patient can benefit from DA approach, and our results are in agreement with what emerged from the prospective randomized study by Komnos et al. [18]; he studied 122 patients treated following a predefined preoperative and postoperative protocol, and identified in the anterior approach, in anti-hemorrhagic prophylaxis, age, BMI and type of anesthesia protective tools against blood loss, capable of reducing the transfusion rate (transfusion rate: DA 5,4% vs. PL 15,2%, *p* value < 0.05). The statistical analysis deriving from the stratification by age groups (< 55 years, 56–65 years, > 75 years) did not yield significant results, but according to the previous study the individual medical circumstances of the patients represent risk factors not controllable and should be evaluated by a multidisciplinary team for the adoption of best precautions.

Independent factors like obesity, chronic therapies with anticoagulant drugs and with antiplatelet drugs have been demonstrated by different authors like Patil et al. [22] and Rosas et al. [23] to be independent risk factors for intraoperative bleeding and the need of transfusions; the choice of the surgical approach is not able to determine a significant reduction of the risk of bleeding.

In agreement with results obtained, use of tranexamic acid, as emerges from the meta-analysis by Komnos et al., drastically reduces blood loss and rate of transfusion; moreover, the safety of this prophylactic treatment [18] is confirmed in the meta-analysis by Moskal et al. [24]. Moreover we can say that in the era of evidence-based medicine, where minimizing patient complications is crucial, the surgical approach is another important factor, under the supervision of the surgeon, to reduce the risk of predictable perioperative complications, such as those related to bleeding [25, 26].

The study is conditioned by some limitations: The main one is related to the method of data collection, carried out retrospectively, which represents a potential patient selection bias. Another limitation is related to the follow-up of patients, limited to the period of hospitalization, effectively losing the possibility of recording events subsequent to the date of discharge.

Starting from our experience, it would be interesting to start a prospective study with more restrictive patient selection criteria, matching patients by age and preoperative clinical conditions, adopting a standardized perioperative surgical and anesthetic protocol, to obtain a comparison as accurate as possible.

## Conclusions

Anterior and posterolateral approach is a valid choice in hip replacement surgery, when minimal invasiveness with abductor muscle preservation is required, to permit a faster rehabilitation with optimal outcomes. To reduce and optimize THA protocol can be suggested the use of premedication with tranexamic acid, because seems to be effective in preventing postoperative bleeding and is therefore advisable in patients with a high risk of bleeding. In addition, patients treated by the anterior approach undergo a significantly shorter hospitalization. From the analysis of patient’s subgroups those aged between 66 and 75 years benefit from the DA approach, with less frequent transfusion requirement.
